# Rare case of pure medial subtalar dislocation in a basketball player

**DOI:** 10.11604/pamj.2016.23.106.8848

**Published:** 2016-03-16

**Authors:** Abdellatif Benabbouha, Nacer Ibou

**Affiliations:** 1 Service de Chirurgie Orthopédique et Traumatologique I, Hôpital Militaire d'Instruction Mohamed V, Rabat, Maroc

**Keywords:** Dislocation, isolated, medial, subtalar

## Image in medicine

Pure acute medial subtalar dislocation without any fractures is very rare and hardly reported in the literature, it represents approximately 1% of all dislocations. This injury is defined as simultaneous dislocation of both thetal on avicular and the talocal caneal joints without a major fracture. It is not commonly seen as a sports injury because it requires transfer of a high energy. Optimal management of subtalar dislocations is immediate closed reduction with procedural sedation. We report a very rare case of a closed subtalar dislocation without any related fractures. A 22 year old male was admitted to the emergency department with pain and ankle deformity following an inversion injury during a basketball game. In his physical examination, the left foot was displaced medially and talus was prominent dorso laterally (A). However, there was not any neurovascular compromise. The X-ray examination revealed medial subtalar dislocation without associated fractures (B, C). A computed tomography scan with 3D reconstruction confirmed the isolated dislocation. Under procedural sedation the reduction was successfully performed by an external maneuver (D). The ankle was immobilized in a short leg cast for 8 weeks. At 24 months follow-up, the patient was autonomous and active without instability at the left ankle.

**Figure 1:**
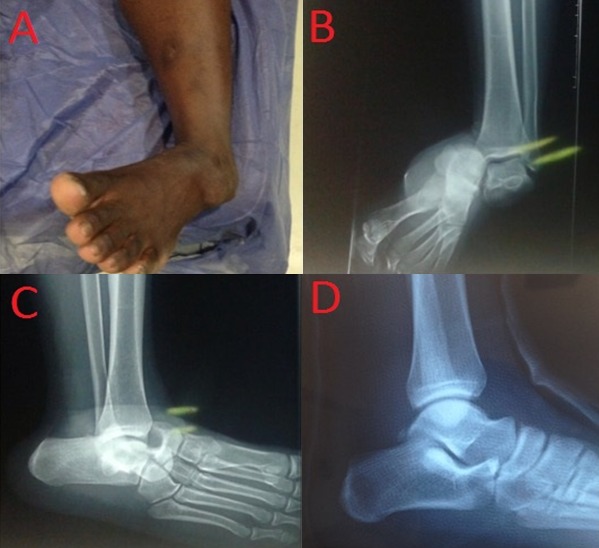
(A) Clinical appearance of a medial subtalar dislocation of the foot; (B, C) radiographs of the left ankle demonstrating a medial subtalar dislocation; (D) radiographs after reduction and immobilization with a posterior splint

